# Image Clustering Algorithm Based on Predefined Evenly-Distributed Class Centroids and Composite Cosine Distance

**DOI:** 10.3390/e24111533

**Published:** 2022-10-26

**Authors:** Qiuyu Zhu, Liheng Hu, Rui Wang

**Affiliations:** School of Communication & Information Engineering, Shanghai University, Shanghai 200444, China

**Keywords:** clustering, composite cosine distance, contrastive learning, predefined evenly-distributed class centroids (PEDCC)

## Abstract

The clustering algorithms based on deep neural network perform clustering by obtaining the optimal feature representation. However, in the face of complex natural images, the cluster accuracy of existing clustering algorithms is still relatively low. This paper presents an image clustering algorithm based on predefined evenly-distributed class centroids (PEDCC) and composite cosine distance. Compared with the current popular auto-encoder structure, we design an encoder-only network structure with normalized latent features, and two effective loss functions in latent feature space by replacing the Euclidean distance with a composite cosine distance. We find that (1) contrastive learning plays a key role in the clustering algorithm and greatly improves the quality of learning latent features; (2) compared with the Euclidean distance, the composite cosine distance can be more suitable for the normalized latent features and PEDCC-based Maximum Mean Discrepancy (MMD) loss function; and (3) for complex natural images, a self-supervised pretrained model can be used to effectively improve clustering performance. Several experiments have been carried out on six common data sets, MNIST, Fashion-MNIST, COIL20, CIFAR-10, STL-10 and ImageNet-10. Experimental results show that our method achieves the best clustering effect compared with other latest clustering algorithms.

## 1. Introduction

Clustering is the process of dividing a collection of physical or abstract objects into classes composed of similar objects. The clusters generated by clustering algorithms are some sample sets. The samples in the same cluster are similar to each other, but different from those in other clusters.

In this paper, an efficient image clustering algorithm based on predefined evenly-distributed class centroids and composite cosine distanc e(ICBPC) is proposed. In this algorithm, PEDCC [1] is used as the clustering centers to ensure the maximum inter-class distance of latent features. PEDCC has been applied to several of our studies, such as classification [2] and out-of-distribution detection [3]. In [2], our contribution is mainly focused on classification tasks with supervised learning. In [3], our contribution is mainly focused on out-of-distribution detection, which is designed to detect test samples with non-overlapping labels relative to training data. Both algorithms are supervised learning algorithms that require the labels of the training data. In this paper, PEDCC is applied to achieve better clustering performance. Clustering is an unsupervised learning method that does not require labels of training data, while classification and out-of-distribution detection are both supervised learning methods. The data distribution constraint and contrastive constraint between samples and augmented samples are applied to improve the clustering performance. The specific training process is to input the samples and their augmented samples into the encoder at the same time to obtain their features. The distance between the two features are reduced through contrastive loss [4]. Maximum mean discrepancy (MMD) [5] losses are used to make the distribution of samples close to the PEDCC distribution (maximizing distribution similarity between latent features and Dirac distribution within classes). Compared with Euclidean distance, cosine distance can be more suitable for the PEDCC-based MMD loss and contrastive loss.

**Figure 1:**
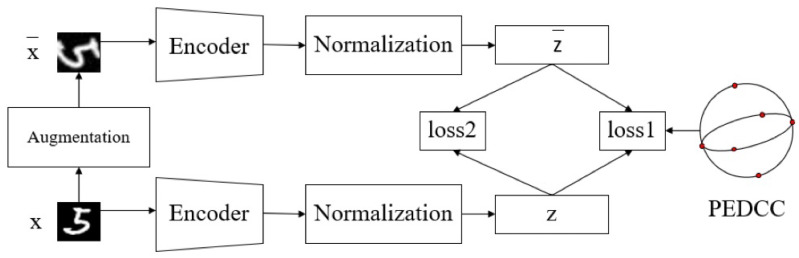
Image clustering network structure. *x* stands for samples, and *z* is encoded latent features of samples, which are used for clustering. The clustering algorithm includes two loss functions.

The algorithm structure is shown in Figure 1. The main contributions of this paper include:

(1) An encoder only clustering network structures is proposed, and PEDCC is used as the clustering center to ensure the maximum inter-class distance in latent feature space. Data distribution constraint and contrastive constraint between samples and augmented samples are applied to improve the clustering performance;
(2) The algorithm normalizes the latent features, and composite cosine distance is proposed to replace Euclidean distance to achieve a better clustering effect. Experiments on several public data sets show that the proposed algorithm achieves the SOTA results.
(3) For complex natural images such as CIFAR-10 and STL-10, a self-supervised pretrained model can be used to effectively improve clustering performance.

In this paper, instead of Euclidean distance, a new composite cosine distance is proposed to better fit the PEDCC clustering model, which has never been proposed before and can be widely used for various image clustering tasks. At the same time, we applied the contrastive loss function to the clustering algorithm and achieved good results. Contrastive learning has previously been used in the field of self-supervised learning. At last, we found that, for complex natural images, a self-supervised pretrained model can be used to effectively improve clustering performance.

The paper is arranged as follows: Section 2 summarizes the related work, and our methods are introduced in detail in Section 3. Then, in Section 4, we give the experimental settings and results. Finally, Section 5 summarizes the whole paper. The code can be downloaded at https://github.com/LihengHu/ICBPC (accessed on 29 August 2022).

## 2. Related Work

### 2.1. Clustering and Deep Learning Based Clustering Method

Clustering is one of the most important unsupervised learning tasks. The purpose of clustering is to classify similar data into a cluster based on some similarity measures. The traditional clustering methods include partition-based method [6] and hierarchical method [7]. The disadvantage of traditional clustering is that the similarity measurement method used is inefficient, and the performance of the traditional clustering method is poor on high-dimensional data, and it has high computational complexity on large-scale data sets. The solution is to reduce and transform features, which maps the original data into a new feature space, making the generated data more easily separated by the existing classifier.

Hierarchical clustering algorithm starts with many small clusters and then gradually merges into large clusters. The partition clustering method minimizes the sum of the squared errors between the data points and their nearest cluster centers. Among them, the k-means [6] algorithm has attracted the most attention. The k-means algorithm takes k as the parameter and divides n objects into k clusters, so that the similarity within the clusters is high, while the similarity between the clusters is low.

In the last few years, deep neural networks have had great success. The success of deep learning often depends on the support of large amounts of data, and the supervised learning of large amounts of data is mature, such as [8,9]. However, it takes a lot of time and resources to mark massive data. Unsupervised learning does not need to rely on data labels, and can automatically discover the latent structure in the data, saving a lot of time and hardware resources.

Auto-encoder (AE) [10,11] is one of the most important algorithms in unsupervised representation learning. Since the dimension of the latent layer is generally smaller than that of the data layer, it can help extract the most salient features of the data. AE is mainly used to find better initializations for parameters in supervised learning and can also be combined with unsupervised clustering. AE can be thought of as consisting of two parts: an encoder that maps the raw data X to represent H, and a decoder that generates the reconstruction.

Deep embedding for clustering (DEC) [12] uses the auto-encoder as the network architecture. First, the auto-encoder is trained by rebuilding the loss, and the decoder part is discarded. The features extracted from the encoder network are used as the input of the clustering module. After that, clustering allocation is used to strengthen the loss to fine-tune the network. At the same time, the clustering is iteratively improved by minimizing the KL divergence between the distribution of soft tags and the distribution of auxiliary targets. Discriminatively boosted image clustering (DBC) [13] has almost the same architecture as DEC, with the only improvement being the use of a convolutional auto-encoder. Its performance on image data sets is superior to DEC due to the use of convolutional networks.

Pseudo-supervised deep subspace clustering (PSSC) [14] based on auto-encoder uses pair similarity measure to reconstruct loss to obtain local structural information, while similarity is a layer of learning through self-expression. Pseudo graphs and pseudo labels can benefit from the uncertain knowledge gained from online training, and are further used to monitor similar learning. Image clustering with deep semantic embedding (DSEC) [15] extracts the total semantic (attribute) features from the data set firstly, and then employs a deep semantic embedding auto-encoder to refine the lower dimensional multi-features representation. The final clustering work is implemented by iteratively optimizing a KL divergence-based clustering objective. Representation learning based on an auto-encoder and deep adaptive clustering for image clustering(RLBAD) [16] presents a novel representation learning method and we use it to solve the image clustering problem. It borrows the deep adaptive image clustering (DAC) [17] algorithm and incorporates it to train a fully convolutional auto-encoder.

The DAC algorithm combines feature learning and clustering. It transforms the clustering problem into a binary pairwise classification framework to judge whether image pairs belong to the same cluster. In DAC, similarity is calculated as the cosine distance between the image label features generated by deep convolutional networks. Our algorithm employs compound cosine distances to fit the PEDCC model.

Associative Deep Clustering [18] is a direct clustering algorithm for deep neural networks. The central idea is to jointly train centroid variables with the network’s weights by using a clustering cost function. Predefined evenly-distributed class centroids are used as the clustering centers to ensure the maximum inter-class distance of latent features in our algorithm. DeepCluster [19] is a clustering method that jointly learns the cluster assignments of neural network parameters and resulting features. DeepCluster uses k-means to iteratively group features and uses subsequent assignments as supervision to update the weights of the network.

An image clustering auto-encoder (ICAE) [20] combines predefined clustering centers with auto-encoders to obtain better results. ICAE differs from our algorithm mainly in the structure, the design of the loss function and the distance measure. Although an auto-encoder can achieve good results, it is complex in structure and requires long training time. The algorithm that we proposed simplifies the structure by using only the encoder and discarding the decoder. At the same time, the performance of our algorithm exceeds that of the algorithm using an auto-encoder.

We compare the experimental results of these algorithms in Section 4.6.

### 2.2. PEDCC

**Figure 2:**
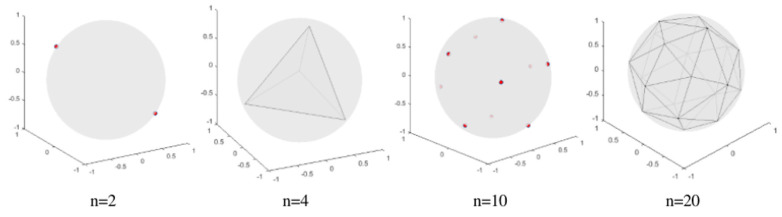
PEDCC points visualization in three-dimensional feature space, where n is the number of predefined class centroids.

Zhu and Zhang proposed the classification supervised auto-encoder (CSAE) [1] to implement the classification function with a unified auto-encoder network structure using the predefined evenly-distribution class centers, and to generate samples of different classes according to the class label. PEDCCs are class center points evenly distributed on the unit hypersphere of the latent feature space, which are used as the training target of the classification network to maximize the inter-class distance. Figure 2 shows PEDCC visual instances. As mentioned above, PEDCCs are some evenly-distributed points on the hypersphere, whose distribution can be regarded as the sum of a set of Dirac functions.

In CSAE, the samples were labeled. In contrast, we use PEDCC for clustering. We learn the mapping function and map the different classes of samples to these predefined class centers, so that different classes can be distinguished by the strong fitting ability and effectiveness of deep learning.

## 3. Methods

In this section, we will introduce the implementation process of the ICBPC algorithm and loss function. Section 3.1 introduces the algorithm process and Section 3.2, Section 3.3 and Section 3.4 introduce the design of the loss function.

### 3.1. ICBPC

The implementation process of ICBPC algorithm is shown as Algorithm 1. First, we perform data augmentation on each unlabeled image *X* to obtain $\hat{X}$. Then, both the original image and the augmented image are input into the encoder to obtain its latent features *Z* and $\hat{Z}$. Then, the distance between the two features are reduced by contrastive loss ($loss_{2}$). MMD [5] ($loss_{1}$) is used to make the distribution close to PEDCC distribution (maximizing distribution similarity between latent features and Dirac distribution within classes). In two loss functions, we replace the Euclidean distance with a composite cosine distance to fit the model.
**Algorithm 1** ICBPC algorithm**Input:***X* = unlabeled images;**Output:***K* classes of clustering images;  1: Initialize PEDCC cluster centers;  2: **repeat**  3:    $\hat{X}$ = Augumentation(*X*);  4:    $\hat{Z}$ = Encoder($\hat{X}$); *Z* = Encoder(*X*);  5:    loss1 = MMD($Z⊙\hat{Z}$, PEDCC); loss2 = Contrastive loss(Z, $\hat{Z}$);  6: **until** Stopping criterion meet

### 3.2. Composite Cosine Distance for Normalized Features and PEDCC

Euclidean distance is generally used to measure the distance in different loss functions. To better fit our PEDCC-based clustering model, we normalized the latent features and then replaced Euclidean distance with composite cosine distance. For Euclidean distance $d^{2}$, we have:

(1) $$d^{2}={\left(x_{1}-x_{2}\right)}^{T}\left(x_{1}-x_{2}\right)=2\left(1-x_{1}^{T}x_{2}\right)=2\left(1-cos\theta \right)$$

where θ is the angle between $x_{1}$,$x_{2}$. In this paper, we use $d_{\theta }$ = 1−cosθ as a new distance metric for all loss functions, that is the original Euclidean distance $d^{2}$ is 2 * $d_{\theta }$.

The cosine distance does not meet the triangle inequality criterion of the conventional distance metric, that is, the sum of the side lengths of the two short sides will be less than the side length of the long side. However, in the training process of our loss function, this property may be a good thing. In the process of gradual iteration between the initial value and the training target, the sum of the cosine distances in each step will be shorter than the cosine distance in one step, which can speed up the convergence, and also be proved by later experiments.

**Figure 3:**
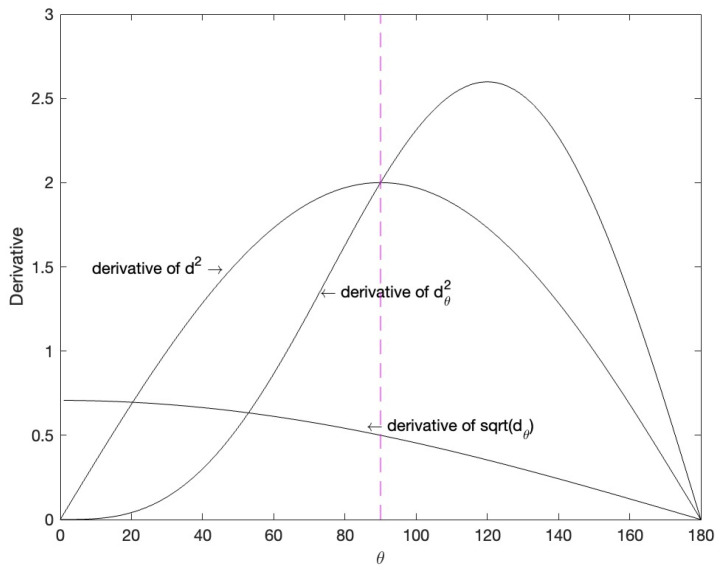
Derivative of $d^{2}$, $d_{\theta }^{2}$ and $\sqrt{d_{\theta }}$ within the range of 0 to $180^{\circ }$. The x-axis is the angle between the features, and the y-axis is the gradient value.

The change of derivative values of $d^{2}$ and $d_{\theta }^{2}$ within the range of 0 to $180^{\circ }$ are shown in the Figure 3. It can be seen from the figure that when θ is greater than $90^{\circ }$, $d_{\theta }^{2}$ has a larger gradient and the training is easier to converge.

To improve the derivative of cosine distance at small angles, we could use $\sqrt{d_{\theta }}$. It can enhance the ability of network parameter updating in the later training period.

The change of derivative values of $\sqrt{d_{\theta }}$ within the range of 0 to $180^{\circ }$ are shown in the Figure 3. It can be seen that with the decrease of the θ angle, the gradient gradually increases, which is conducive to the network update in the later stage of training, and avoids the problem that the gradient of $d_{\theta }^{2}$ gradually tends towards zero.

**Figure 4:**
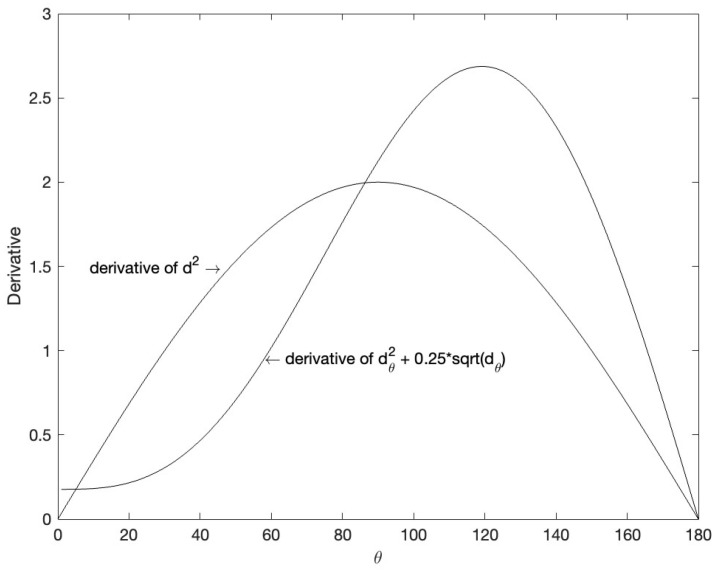
Derivative of composite cosine distance $\;d_{\theta }^{2}+0.25\sqrt{d_{\theta }}$ and Euclidean distance $d^{2}$ within the range of 0 to $180^{\circ }$. The x-axis is the angle between the features, and the y-axis is the gradient value.

In our two loss functions, $d^{2}$ is necessary in this paper, replaced by composite cosine distance $d_{c}^{2}$ = $d_{\theta }^{2}+\alpha \sqrt{d_{\theta }}$. α is set to 0.25 and the value of α comes from the experiment. The change of derivative values of $d_{\theta }^{2}+\alpha \sqrt{d_{\theta }}$ within the range of 0 to $180^{\circ }$ are shown in the Figure 4. It can be seen from the figure that when θ is greater than $90^{\circ }$, the new distance has a larger gradient and the training is easier to converge, and when θ is small, the gradient is still greater than zero to strengthen the training of the small angle. Experiments show that this distance can obtain a better clustering effect compared with Euclidean distance.

### 3.3. Clustering Loss Function

The loss function based on PEDCC utilizes the concept of PEDCC in CSAE network to set PEDCCs as the clustering centers of classes, and these clustering centers are evenly-distributed on the hypersphere of feature space, maximizing the inter-class distance and obeying Dirac distribution within the class. Our algorithm uses MMD to measure the distance between the samples’ distribution and PEDCC distribution. The basic principle of the MMD is to find a function that assumes that two different distributions have different expectations. If the function is evaluated with empirical samples from the distribution, the function will indicate whether they are from different distributions. Our $loss_{1}$ aims to utilize the distribution difference between the samples’ distribution and PEDCC distribution in latent features, so that the features extracted from the encoder meet the distribution of PEDCC.

The MMD algorithm is used as $loss_{1}$ to train the network, and the formula is as follows:

$$loss_{1}=MMD\left(\left[Z,\hat{Z}\right],PEDDC\right)$$

$$=\frac{1}{M(M-1)}\overset{M}{\underset{i\neq j}{\sum }}k\left(l_{i},l_{j}\right)+\frac{1}{c(c-1)}\overset{C}{\underset{i\neq j}{\sum }}k\left(u_{i},u_{j}\right)$$

(2) $$-\frac{2}{MC}\overset{M,C}{\underset{i,j=1}{\sum }}k\left(l_{i},u_{j}\right).$$

where *Z* is the intermediate latent features, $\hat{Z}$ means the latent features of the augmented data, *M* means its dimension, $l_{i}=\left[Z,\hat{Z}\right]$ is the latent features of the image and its augmented latent features; $u_{i}$ represents the PEDCC class centers, *C* is its number, and k(x,y) is the kernel function.

By iteratively minimizing $loss_{1}$, the probability distribution of latent features can be closer to that of PEDCC. The underlying features are also going to be close to these points on the hypersphere.

The kernel function $k(x_{1},x_{2})$ is usually expressed in the form of radial basis function, and its value is inversely proportional to the square of the distance between $x_{1}$ and $x_{2}$. The formula of the kernel function is as follows:

(3) $$k\left(x_{1},x_{2}\right)=e^{\frac{-d_{c}^{2}}{2\sigma^{2}}}.$$

where composite cosine distance replaces Euclidean distance $d^{2}$.

$Loss_{1}$ uses the MMD algorithm based on a radial basis to make the latent feature distribution the same as the predefined PEDCC, achieving the best clustering. In $loss_{1}$, cosine distance is used to better measure the distance between two features, which makes the radial basis-based MMD algorithm easier to converge.

### 3.4. Data Augmentation Loss Function

The main purpose of data augmentation is to reduce the overfitting of the network and help the network extract more discriminative features. By transforming the training images, a network with a stronger generalization ability can be obtained, which can better adapt to the application scenarios.

We use some common data augmentation. One type of augmentation involves spatial and geometric transformation of data, such as cropping, resizing (with horizontal flipping) and rotation [21]. The other type of augmentation involves appearance transformation, such as color distortion (including color dropping, brightness, contrast, saturation) [22], Gaussian blur, and Sobel filtering.

**Figure 5:**
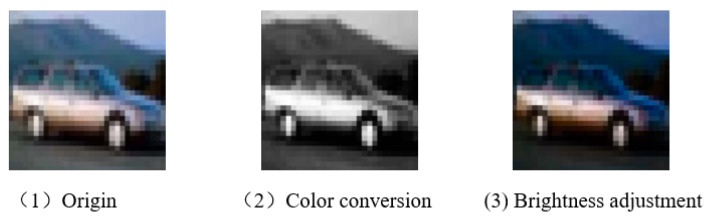
Data augmentation of CIFAR10.

**Figure 6:**
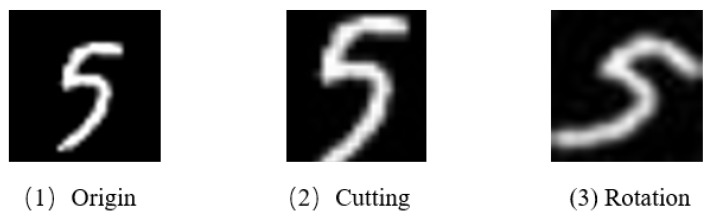
Data augmentation of MNIST.

For different datasets, we should adopt different data augmentation methods to get better clustering effect for datasets. For example, for the color image datasets, we mostly adopt color conversion, brightness adjustment and other methods, as shown in Figure 5. However, geometric processing are used such as cutting and rotation, as shown in Figure 6, to achieve better clustering effect for MNIST.

The samples $\hat{X}$ augmented by unlabeled data *X* are input into the encoder to obtain the features $\hat{Z}$ and *Z*, which can be used to achieve better clustering.

Contrastive loss function is used to constrain the features of the augmented samples and the features of the original samples.

Contrastive loss is mainly used for dimensionality reduction, that is, after dimensionality reduction (feature extraction) of the originally similar samples, the two samples are still similar in the feature space. However, after dimensionality reduction for the originally dissimilar samples, the two samples are still dissimilar in the feature space. Similarly, the loss function can well express the matching degree of the samples.

The contrastive loss function has the following expression:

(4) $$loss_{2}\left(x_{1},x_{2},y\right)=\frac{1}{2N}\overset{N}{\underset{i=0}{\sum }}\left[yd^{2}+\left(1-y\right)max{\left(margin-d,0\right)}^{2}\right]$$

where *d* represents the distance of the features of the two samples, $x_{1}$ represents the original sample, $x_{2}$ represents the augmented sample or random negative sample. *y* represents the label of whether the two samples match or not, *y* = 1 represents the similarity or match of the two samples, *y* = 0 represents the mismatch, and margin is the set threshold. N is the number of sample pairs. Margin is usually set to 0.3.

As mentioned above, $d^{2}$ is also replaced by $d_{c}^{2}$ in Equation (4). Formula is as follows:

(5) $$loss_{2}\left(x_{1},x_{2},y\right)=\frac{1}{2N}\overset{N}{\underset{i=0}{\sum }}\left[yd_{c}^{2}+\left(1-y\right)max{\left(margin-d_{c},0\right)}^{2}\right]$$

When $x_{2}$ is the augmented sample, *y* = 1 (that is, the samples are similar). If the distance in the feature space is large, it indicates that the current model is not good, so the loss is increased.

When $x_{2}$ is the random negative sample, *y* = 0 (the samples are not similar). If the samples are not similar and the distance is small, the loss value will increase.

$Loss_{2}$ expects that the cosine distance of the augmented samples in the latent feature space is the minimum to achieve correct clustering. In $loss_{2}$, the cosine distance also replaces Euclidean distance, so that the original and augmented samples have the same direction, rather than the same value.

### 3.5. Loss Function

The loss function of the whole algorithm is combined with the above two loss functions, as follows:

(6) $$loss={loss}_{1}+\lambda \times {loss}_{2}.$$

where λ is the weight of $loss_{2}$. For different data sets, the weights of the two loss functions will be adjusted, and different weights will lead to different results. The weights are shown in Table 1. For the kernel function of MMD loss, *µ* is set to 2.0 and kernel number is set to 5.0 in our experiments.

### 3.6. Using Self-Supervised Pretrained Model

Self-supervised pretrained model is a network that is trained on a large amount of data by self-supervised learning. Since the pretraining model can bring up more effective image features, further implementation of clustering algorithm on the pretraining model can make the algorithm obtain more discriminative features, and achieve better clustering performance, especially for complex natural images, such as CIFAR-10, STL-10 and ImageNet-10. In the experiments, we use the typical Barlow Twins [23] self-supervised learning algorithm to pretrain the ResNet model on the Imagenet.

## 4. Experiments and Discussions

### 4.1. Experiments Settings

#### 4.1.1. Datasets

We used six datasets to verify the performance of our algorithm. The six datasets are MNIST, COIL20, FASHION-MNIST, CIFAR-10, STL-10, and ImageNet-10 as Table 2. We randomly choose 10 subjects from the ImageNet dataset to construct the ImageNet-10 dataset for our experiments. All datasets before inputting the network are normalized to [−1, 1].

#### 4.1.2. Experimental Setup

Before starting the experiment, we set the number of classes of classification and the dimension of middle layer features. Set the initial learning rate to 0.001 and use the Adam optimizer. The batch-size is set to 100 and the training epoch is 400. The network structure keeps unchanged during the training. The settings of hyper-parameters are shown in Table 1. The values in Table 1 are set when the clustering results are the best. The value of λ is set differently for the six different datasets. Setting the value of λ to 8 achieves the best clustering results for MNIST, Fashion MNIST, STL-10 and ImageNet-10. When the value of λ is set to 9, the best clustering results can be obtained for COIL20 and CIFAR-10. All our experimental results are averaged after 4 times of training.

#### 4.1.3. Evaluation Metrics

We use the following two indicators to validate our algorithm: Cluster Accuracy (ACC) [24] and Normalized Mutual Information (NMI) [24].

#### 4.1.4. Encoder Architecture

ResNet [25] can solve the problem of deep neural network degradation. So, our algorithm uses the residual network structure ResNet-18 as the encoder, and the specific network structure of the encoder is shown in Table 3.

For CIFAR-10, STL-10, and ImageNet-10, we adopt a self-supervised pretrained ResNet model trained on the ImageNet dataset. The network only trains the last two blocks, and the parameters of the other parts are frozen.

The dimension of the latent feature of the middle layer is the dimension of the predefined class center. The dimension of the middle layer is different for different datasets and can be determined according to the experiment. Taking MNIST as an example, the performance of the models in different dimensions is shown in Table 4. Other datasets also obtain the best latent features dimension through experiments.

The best dimensions of the latent features used for each dataset are shown in Table 5. It can obtain the best model performance. Through training, the distribution of latent feature *Z* can be close to the PEDCC distribution.

### 4.2. Analysis on Computational Time and Clustering

**Figure 7:**
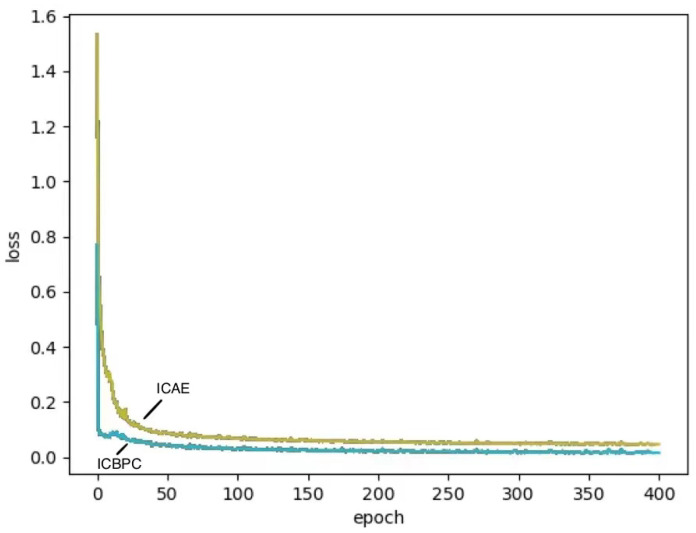
Change of loss value with epoch.

We used the PyTorch deep learning framework to do all the training on an Inter(R) I7-6700K CPU, 32GB RAM, and a Nvidia GTX 1080 TI GPU. There are two loss functions in total, and the convergence time is fast. Taking the COIL20 dataset as an example, only 14 s are needed for each epoch, achieving the highest accuracy within 400 epochs. It only requires 4 s to obtain ACC and NMI for network testing. The proposed composite cosine distance can significantly improve the convergence speed. The change of loss value with epoch is shown in Figure 7, which shows that our algorithm converges faster than the ICAE algorithm.

**Figure 8:**
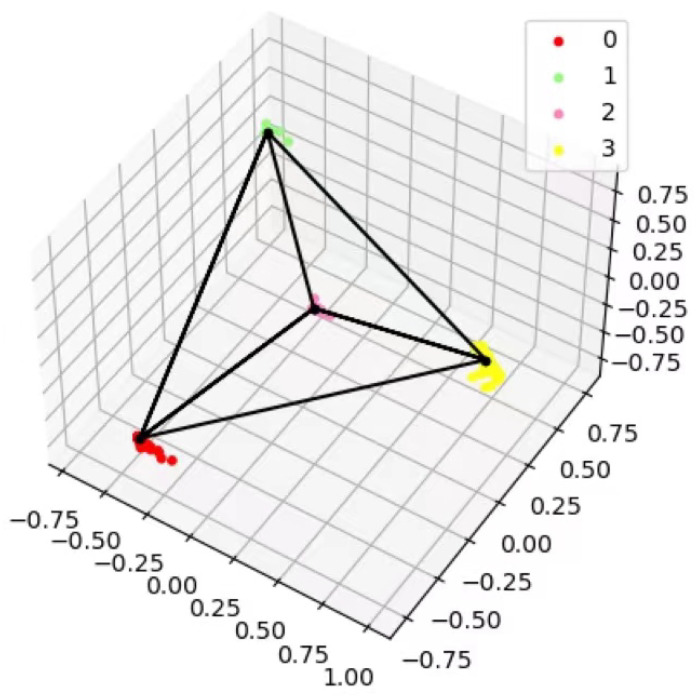
3D feature visualization.

To demonstrate the clustering effectiveness of our model, we select four classes of the MNIST and set the feature dimension to 3 for training. As shown in Figure 8, we visualize the resulting features in 3D coordinates. It can be seen from the figure that the distance between each category is far enough.

### 4.3. Ablation Experiment

We tested the effectiveness of each loss function with some ablation experiment. Experimental results are shown in Table 6, which shows that the best clustering effect can be obtained by using the two loss functions and composite cosine distance.

### 4.4. Effectiveness of Self-Supervised Pretrained Model

For CIFAR-10, STL-10, and ImageNet-10, we adopt self-supervised pretrained ResNet model trained on the ImageNet. We resize STL-10 to 224 × 224 × 3 to fit the pretrained model. The network only trains the last two blocks, and the parameters of other parts are frozen. As shown in Table 7, a self-supervised pretrained model can be used to effectively improve the clustering performance for complex natural images. The clustering performance of Fashion-Mnist is not improved by the pretrained model. It can be seen that the pretrained model is more effective for complex natural images.

### 4.5. Compared with Auto-Encoder

The algorithm that we proposed simplifies the algorithm structure by using only the encoder and discarding the decoder. At the same time, the performance of our algorithm exceeds that of the algorithm using the auto-encoder. We compared the two structures, and the results are shown in Table 8. The encoder-only model has shorter training time and higher accuracy.

### 4.6. Compared with the Latest Clustering Algorithm

We compared the ICBPC clustering algorithm with the latest clustering algorithm, and our algorithm achieved excellent results in all four datasets, as shown in Table 9.

In Table 9, all the results are reported by running the code they posted or are taken from the corresponding paper. The mark “-” means that the result is not available for the paper or code. The significance of bold in the Table 9 represents the best result.

Compared with deep clustering algorithms using auto-encoders such as DCN and DEN, our model is simpler in structure, faster in training, and can achieve good clustering performance by PEDCC. Compared with other algorithms that learn feature representations for clustering such as JULE, our algorithm uses PEDCC to make the inter-class distances large enough for better clustering performance.

### 4.7. Statistical Analysis of Experimental Data

All our experimental results are averaged after 4 times of training. We calculate the standard deviation of the experimental data to verify the stability of the algorithm. As shown in Table 10, the standard deviation values of the experimental results are low, which can prove the stability of our algorithm.

## 5. Conclusions

This paper presents an image clustering algorithm based on predefined evenly-distributed class centroids and composite cosine distance. In this algorithm, an encoder only network structure is adopted and PEDCC is used as the clustering center to ensure the maximum distance between classes of latent features. Data distribution constraints and contrastive constraints between samples and augmented samples are applied to improve the clustering performance. We use composite cosine distance instead of Euclidean distance to better fit the PEDCC model. This algorithm achieves better performance than the existing clustering algorithms on MNIST, COIL20, Fashion-MNIST, CIFAR-10, STL-10 and ImageNet-10. For complex natural images, a self-supervised pretrained model is used to achieve better clustering performance. In the future, we will continue to use the characteristics of PEDCC for feature representation learning, to obtain better clustering and recognition results.

## Figures and Tables

**Table 1:** Hyper-parameters setting of our algorithm. The value of the setting is obtained by experiment.

Datasets	λ	α
MNIST	8.00	0.25
COIL20	9.00	0.25
Fashion MNIST	8.00	0.25
CIFAR-10	9.00	0.25
STL-10	8.00	0.25
ImageNet-10	8.00	0.25

**Table 2:** Datasets.

Datasets	Samples	Categories	Image Size
MNIST	70,000	10	28 × 28
COIL20	1440	20	128 × 128
Fashion-MNIST	70,000	10	28 × 28
CIFAR-10	60,000	10	32 × 32 × 3
STL-10	5000	10	96 × 96 × 3
ImageNet-10	13,000	10	224 × 224 × 3

**Table 3:** Network structure of the encoder.

Layer	Output Size	Remarks
Conv1	32×32	32 channels
maxpool	32×32	3×3, stride = 2
BasicBlock1	16×16	64 channels
BasicBlock2	8×8	128 channels
BasicBlock3	4×4	256 channels
BasicBlock4	2×2	512 channels, Encoder output
Fully connected layer 1	dimension of latent features	latent features

**Table 4:** Model performances in different dimensions. The significance of bold represents the best result.

Data Sets	Dimension of Latent Features	ACC	NMI
MNIST	40	0.986	0.979
MNIST	60	**0.994**	**0.985**
MNIST	80	0.989	0.980
MNIST	100	0.982	0.976

**Table 5:** Dimension of latent features. The value of the setting is obtained by the experiment.

Datasets	Dimension of Latent Features
MNIST	60
COIL20	160
Fashion MNIST	100
CIFAR-10	60
STL-10	100
ImageNet-10	100

**Table 6:** Ablation experiment results. The significance of bold represents the best result.

Datasets	${\mathrm{Loss}}_{1}$	${\mathrm{Loss}}_{2}$	Composite Cosine Distance	Euclidean Distance	Normal Cosine Distance	ACC	NMI
MNIST	✓		✓			0.398	0.312
MNIST	✓	✓	✓			**0.994**	**0.985**
MNIST	✓	✓		✓		0.981	0.961
MNIST	✓	✓			✓	0.982	0.965
Fashion-MNIST	✓		✓			0.467	0.354
Fashion-MNIST	✓	✓	✓			**0.737**	**0.714**
Fashion-MNIST	✓	✓		✓		0.725	0.699
Fashion-MNIST	✓	✓			✓	0.722	0.693
COIL20	✓		✓			0.410	0.561
COIL20	✓	✓	✓			**0.960**	**0.982**
COIL20	✓	✓		✓		0.920	0.960
COIL20	✓	✓			✓	0.920	0.958
CIFAR-10	✓		✓			0.124	0.113
CIFAR-10	✓	✓	✓			**0.298**	**0.182**
CIFAR-10	✓	✓		✓		0.278	0.172
CIFAR-10	✓	✓			✓	0.273	0.163
STL-10	✓		✓			0.186	0.157
STL-10	✓	✓	✓			**0.551**	**0.525**
STL-10	✓	✓		✓		0.535	0.519
STL-10	✓	✓			✓	0.540	0.522
ImageNet-10	✓		✓			0.152	0.234
ImageNet-10	✓	✓	✓			**0.412**	** 0.375**
ImageNet-10	✓	✓		✓		0.401	0.349
ImageNet-10	✓	✓			✓	0.405	0.356

**Table 7:** Performances using pretrained models. The significance of bold represents the best result..

Datasets	Without Pretrained	Pretrained	ACC	NMI
Fashion-MNIST	✓		**0.714**	**0.737**
Fashion-MNIST		✓	0.712	0.732
CIFAR-10	✓		0.241	0.125
CIFAR-10		✓	**0.298**	**0.182**
STL-10		✓	0.293	0.205
STL-10	✓		**0.551**	**0.525**
ImageNet-10		✓	0.250	0.193
ImageNet-10	✓		**0.412**	**0.375**

**Table 8:** Performances comparison between two structures. The significance of bold represents the best result.

Datasets	Encoder-Only	Auto-Encoder	Training Time of Each Epoch (s)	ACC	NMI
MNIST		✓	58	0.988	0.965
MNIST	✓		40	**0.994**	**0.985**
Fashion-MNIST		✓	122	0.689	0.731
Fashion-MNIST	✓		75	**0.714**	**0.737**
COIL20		✓	29	0.920	0.953
COIL20	✓		14	**0.960**	**0.982**
CIFAR-10		✓	132	0.284	0.163
CIFAR-10	✓		98	**0.298**	**0.182**
STL-10		✓	86	0.532	0.521
STL-10	✓		66	**0.551**	**0.525**
ImageNet-10		✓	205	0.407	0.365
ImageNet-10	✓		130	**0.412**	**0.375**

**Table 9:** Performances comparison with other clustering algorithms.

-	ARCH	NMI	ACC	NMI	ACC	NMI	ACC	NMI	ACC	NMI	ACC	NMI	ACC
-	-	mnist	mnist	coil20	coil20	fashion	fashion	cifar-10	cifar-10	stl-10	stl-10	image-net-10	image-net-10
k-means [6]	-	0.500	0.532	-	-	0.512	0.474	0.064	0.199	0.125	0.192	-	-
SC-NCUT [26]	-	0.731	0.656	-	-	0.575	0.508	-	-	-	-	-	-
SC-LS [27]	-	0.706	0.714	-	-	0.497	0.496	-	-	-	-	-	-
NMF-LP [28]	-	0.452	0.471	-	-	0.425	0.434	0.051	0.180	-	-	-	-
AC-Zell [29]	-	0.017	0.113	-	-	0.100	0.010	-	-	-	-	-	-
AC-GDL [30]	-	0.017	0.113	-	-	0.010	0.112	-	-	-	-	-	-
RCC [31]	-	0.893	-	-	-	-	-	-	-	-	-		
DCN [32]	MLP	0.810	0.830	-	-	0.558	0.501	-	-	-	-		
DEC [12]	MLP	0.834	0.863	-	-	0.546	0.518	0.057	0.208	0.276	0.359		
IDEC [33]	-	0.867	0.881	-	-	0.557	0.529	-	-	-	-	-	-
CSC [34]	-	0.755	0.872	-	-	-	-	-	-	-	-	-	-
VADE [35]	VAE	0.876	0.945	-	-	0.630	0.578	-	-	-	-	-	-
JULE [36]	CNN	0.913	0.964	-	-	0.608	0.563	-	-	0.182	0.277	-	-
DBC [13]	CNN	0.917	0.964	-	-	-	-	-	-	-	-	-	-
DEPICT [37]	CNN	0.917	0.965	-	-	0.392	0.392	-	-	-	-	-	-
CCNN [38]	CNN	0.876	-	-	-	-	-	-	-	-	-	-	-
DEN [39]	MLP	-	-	0.870	0.724	-	-	-	-	-	-	-	-
NC [40]	MLP	-	0.966	-	-	-	-	-	-	-	-	-	-
UMMC [41]	DBN	0.864	-	0.891	-	-	-	-	-	-	-	-	-
TAGNET [42]	-	0.651	0.692	0.927	0.899	-	-	-	-	-	-	-	-
IMSAT [43]	MLP	-	0.983	-	-	-	-	-	-	-	-	-	-
PSSC [14]	AE	0.768	0.843	0.978	**0.972**	-	-	-	-	-	-	-	-
DAC [17]	-	0.935	0.978	-	-	-	-	**0.396**	**0.522**	0.366	0.469	-	-
ADC [18]	-	-	0.987	-	-	-	-	-	0.293	-	-	-	-
ICAE [20]	AE	0.967	0.988	0.953	0.920	0.689	0.731	0.080	0.215	-	-	-	-
ICBPC(ours)	-	**0.985**	**0.994**	**0.982**	0.960	**0.714**	**0.737**	0.182	0.298	**0.525**	**0.551**	**0.412**	**0.375**

**Table 10:** Statistical analysis of experimental results.

Datasets	Average of ACC	Average of NMI	Standard Deviation of ACC	Standard Deviation of NMI
MNIST	0.994	0.985	0.0048	0.0034
COIL20	0.960	0.982	0.0005	0.0013
Fashion MNIST	0.737	0.714	0.0036	0.0041
CIFAR-10	0.298	0.182	0.0045	0.0032
STL-10	0.551	0.525	0.0062	0.0053
ImageNet-10	0.412	0.375	0.0064	0.0055

## Data Availability

The code can be downloaded at https://github.com/LihengHu/ICBPC (accessed on 29 August 2022).
